# A novel ACE inhibitory peptide from *Pelodiscus sinensis* Wiegmann meat water-soluble protein hydrolysate

**DOI:** 10.1007/s00726-024-03399-1

**Published:** 2024-06-07

**Authors:** Pengying Liao, Huayu Liu, Xueqin Sun, Xinrui Zhang, Miao Zhang, Xianyou Wang, Jun Chen

**Affiliations:** 1 College of Pharmacy, Guangxi University of Chinese Medicine, Nanning, 530200 Guangxi China; 2 Guangxi Key Laboratory of Efficacy Study on Chinese Materia Medica, Guangxi University of Chinese Medicine, Nanning, 530200 Guangxi China; 3 School of Pharmacy, Henan University, Kaifeng, 475004 Henan China; 4 Guangxi Key Laboratory of Zhuang and Yao Ethnic Medicine, Guangxi University of Chinese Medicine, Nanning, 530200 Guangxi China; 5 Teaching Experiment and Training Centre, Guangxi University of Chinese Medicine, Nanning, 530200 China

**Keywords:** *Pelodiscus sinensis* Wiegmann, Active peptides, ACE inhibitory activities, Bioinformatics

## Abstract

*Pelodiscus sinensis* meat is a nutritional food and tonic with angiotensin-converting enzyme (ACE) inhibitory activities. To identify the bioactive substances responsible, several bioinformatics methods were integrated to enable a virtual screening for bioactive peptides in proteins identified within a water-soluble protein fraction of *Pelodiscus sinensis* meat by Shotgun proteomics. The peptides were generated from the identified proteins by in silico proteolysis using six proteases. A comparison of the numbers of proteins suitable for digestion with each enzyme and the iBAQ (intensity-based absolute quantification) values for these proteins revealed that bromelain and papain were the most suitable proteases for this sample. Next, the water solubility, toxicity, and ADMET (absorption/distribution/metabolism/excretion/toxicity) properties of these peptides were evaluated in silico. Finally, a novel ACE inhibitory peptide IEWEF with an IC_50_ value of 41.33 µM was identified. The activity of the synthesized peptide was verified in vitro, and it was shown to be a non-competitive ACE inhibitor. Molecular docking revealed that IEWEF could tightly bind to C-ACE, and N-ACE with energies less than 0 kJ mol^−1^, and the peptide IEWEF can form hydrogen bonds with C-ACE and N-ACE respectively. These results provide evidence that bioactive peptides in the water-soluble protein fraction account for (at least) some of the ACE inhibitory activities observed in *Pelodiscus sinensis* meat. Furthermore, our research provides a workflow for the efficient identification of novel ACE inhibitory peptides from complex protein mixtures.

**Supplementary Information:**

The online version contains supplementary material available at 10.1007/s00726-024-03399-1.

## Introduction

Hepatic fibrosis (HF) typically develops alongside the liver tissue repair and reconstruction processes that follow chronic liver injury, irrespective of its etiology. The cellular and molecular mechanisms underlying HF progression include parenchymal cell regeneration, interstitial cell activation, and extracellular matrix (ECM) deposition. Its importance is underlined by the finding that HF is the common pathological basis of most chronic liver diseases, and further deterioration may lead to liver cirrhosis or even liver cancer (Damiris et al. 2020). According to Global Cancer Statistics 2020, liver cancer is the second leading cause of death worldwide (Cao et al. 2021). Hence, prevention of HF progression and its treatment are an important research focus.

The renin-angiotensin system (RAS) also plays an important role in the occurrence and development of HF (Miranda and Simoes e Silva 2017). Angiotensin II (Ang II), angiotensin converting enzyme (ACE), and Ang II type 1 receptor (AT_1_R) together constitute the ACE-Ang II-AT_1_R pathway, which promotes the development of HF. A second pathway comprised of angiotensin converting enzyme 2 (ACE2), Ang(l-7) metabolite (i.e., the product of ACE2 degradation of Ang II), and the Ang(l-7)-binding Mas receptor, which together constitute the ACE2-Ang(l-7)-Mas pathway, has a reverse regulatory effect on the ACE-Ang II-AT_1_R pathway (de Macêdoa et al. 2014). While ACE inhibitors such as captopril, lisinopril, and enalapril are known to regulate blood pressure by affecting the central RAS, they can also relieve organ fibrosis by affecting the local RAS (de Macêdoa et al. 2014). However, long-term use of these ACE inhibitors may elicit adverse reactions, such as dry cough, rash, and tachycardia (Ohishi et al. 2001), and occasionally serious adverse reactions such as renal dysfunction, increased risk of lung cancer, and systemic hypertension (Lin et al. 2020). Therefore, the search for novel ACE inhibitors for the safe treatment of these (and other) diseases is an important research objective.

The conventional strategy of searching for ACE inhibitory peptides in specific proteins is complex and time-consuming. In general, various purification technologies, e.g., ultrafiltration, gel chromatography, anion-exchange chromatography, or high performance liquid chromatography (HPLC), are applied in conjunction with activity evaluations to purify the active fractions (Chen et al. 2021). In recent years, bioinformatics methods have been increasingly utilized for the screening of active peptides (Tian et al. 2022). Proteomics databases, such as UniProt (www.uniprot.org), PDB (www.rcsb.org), and BIOPEP-UWM (https://biochemia.uwm.edu.pl/biopep/start_biopep.php) can be used to obtain the amino acids sequences of a protein of interest (Arámburo-Gálvez et al. 2022; Tian et al. 2022). Peptide cutter (http://www.expasy.org/resources/peptidecutter) or the “Enzymes action” tab in BIOPEP-UWM can then be used to hydrolyze the protein in silico (Arámburo-Gálvez et al. 2022). To evaluate the biological activities of the identified peptides, both Peptide Ranker (http://distilldeep.ucd.ie/PeptideRanker/) and the “Profiles of potential biological activity” tab in BIOPEP-UWM can be subsequently used (Lin et al. 2023; Tian et al. 2022). In addition, ADMETlab can be used to systematically predict the in vivo pharmacokinetic properties (including absorption, distribution, metabolism, excretion, and toxicity) of the active peptides, providing evaluations that are especially conducive to the preliminary screening of the peptides (Dong et al. 2018; Lin et al. 2023). By combining the above bioinformatics tools, it is possible to greatly reduce the complexity of the workflow required for screening ACE inhibitory peptides and decrease the cost of searching for active peptides significantly while improving overall efficiency. However, most of the virtual screenings reported to date have utilized the amino acids sequences of a few specific proteins found in raw materials of interest or have utilized the sequences of proteins containing previously identified peptides (Arámburo-Gálvez et al. 2022; Tian et al. 2022).

*Pelodiscus sinensis* (Wiegmann) is an edible aquatic animal that is nutritionally rich in protein (Wang et al. 2021). It has been demonstrated in numerous reports that protein hydrolysates from different parts of *Pelodiscus sinensis*, including the shell, meat, and egg, exhibit ACE inhibitory activities (Liao et al. 2018; Liu et al. 2012; Pujiastuti et al. 2017). While several ACE inhibitory peptides have been identified from the shell and egg (Liao et al. 2018; Pujiastuti et al. 2017; Rawendra et al. 2013, 2014), none have thus far been identified from the meat. In our previous study, we optimized a process for the enzymatic hydrolysis of water-soluble protein isolated from *Pelodiscus sinensis* meat (WSPM). Using degree of hydrolysis and ACE inhibitory activity as indices, we chose papain as the most suitable protease for this process (Sun et al. 2022). In the present study, an in silico hydrolysis of all proteins in the WSPM fraction was performed on sequences downloaded from UniProt following their identification from whole proteomic data obtained using Shotgun technology. Again, papain was chosen as one of the most suitable proteases, and this was partly consistent with our previous research (Sun et al. 2022). To screen out the ACE inhibitory peptides from WSPM proteins, an array of bioinformatics tools were combined to produce an effective workflow. Following their identification in silico, the active peptides were synthesized and their ACE inhibitory activities were evaluated in vitro by us. The inhibitory mechanisms of proven active peptides were also analyzed by enzyme inhibition kinetics and molecular docking. In summary, we report herein the development of a strategy to screen protein hydrolysates using the most suitable protease, and we have used this strategy to reveal peptides with ACE inhibitory activities in *Pelodiscus sinensis* meat, thus laying a foundation for the further development of *Pelodiscus sinensis* meat protein products.

## Materials and methods

### Materials and chemicals

WSPM prepared in our previous work (Sun et al. 2022) and stored at − 20 °C after freeze-drying was used for this study. ACE (1U, derived from rabbit lung, CAS 207386-83-2), Hippuryl-L-histidyl-L-leucine (HHL, CAS 207386-83-2), and hippuric acid (HA, CAS 495-69-2) were obtained from Sigma-Aldrich Co. (St. Louis, MO, USA). The reagents used for HPLC analysis were all HPLC grade, and all other reagents used were analytical grade. The amounts of each protein were quantified using the BCA Protein Assay Kit (Bio-Rad, USA). All the protein samples were packed separately and stored at − 80 °C.

### Protein extraction and digestion

WSPM samples (20 µg) were mixed with a fivefold volume of SDT buffer (4% SDS, 100 mM Tris-HCl, 1 mM DTT, pH 7.6) and boiled for 5 min. The individual proteins were then resolved on a 12.5% SDS-PAGE gel (constant current 14 mA, 90 min). The resulting protein bands were visualized by Coomassie Blue R-250 staining. Protein digestions were performed using trypsin, and the samples were then desalted on C18 Cartridges (Empore^™^ SPE Cartridges C18, 0.7 × 10 cm, Sigma), concentrated by vacuum centrifugation, and reconstituted in 40 µL of 0.1% (v/v) formic acid.

### LC–MS/MS analysis

LC–MS/MS analyses were performed on a Q Exactive mass spectrometer (Thermo Scientific) coupled to an Easy nLC (Thermo Fisher Scientific). First, the peptide hydrolysates were loaded onto a reverse phase trap column (Thermo Scientific Acclaim PepMap100, 100 µm × 2 cm, 5 µm) connected to a C18-reversed phase analytical column (Thermo Scientific Easy Column, 75 µm × 10 cm, 3 µm) equilibrated in buffer A (0.1% formic acid). The peptides were then separated using a linear gradient of buffer B (84% acetonitrile and 0.1% formic acid) at a flow rate of 300 nL/min (controlled by IntelliFlow technology) over a 120 min run.

The mass spectrometer was operated in positive ion mode. All MS data were acquired using a data-dependent top10 method for dynamically choosing the most abundant precursor ions from the survey scan (300–1800 m*/z*) for HCD fragmentation. The Automatic gain control (AGC) target was set to 3e6, and the maximum inject time to 10 ms. The Dynamic exclusion duration was 40.0 s. Survey scans were acquired at a resolution of 70 000 at *m/z* 200 and the resolution for HCD spectra was set to 17 500 at *m/z* 200, with an isolation width of 2 m*/z*. The Normalized collision energy was 27 eV, and the underfillratio, which specifies the minimum percentage of the target value likely to be reached at maximum fill time, was defined as 0.1%. The instrument was run with peptide recognition mode enabled.

### Identification and quantitation of proteins

The MS raw data for each sample were combined and searched using MaxQuant software 1.5.3.17 (Max Planck Institute of Biochemistry, Martinsried, Germany) against a *Pelodiscus sinensis* database. The parameter settings were as follows: (1) the enzyme used was trypsin and up to two missing cleavages were allowed; (2) fixed modifications was set to carbamidomethyl (C) and variable modifications was set to oxidation (M); (3) only proteins meeting a false discovery rate (FDR) ≤ 0.01 were classified as successfully identified; (4) protein quantification was calculated by the MaxQuant software using intensity-based absolute quantification (iBAQ), which provides an approximation to the absolute concentration of the protein in the sample.

### In silico hydrolysis of WSPM

The amino acids sequences of 49 different proteins identified by Shotgun analysis were obtained from the UniProt database (https://www.uniprot.org/) (July 19, 2023) (The UniProt Consortium 2023). The enzymes chymotrypsin (EC 3.4.21.1), trypsin (EC 3.4.21.4), pepsin (EC 3.4.23.1), papain (EC 3.4.22.2), bromelain (EC 3.4.22.32), and alkaline (EC 3.4.21.62) were selected to hydrolyze these proteins by adopting the “enzyme action” tool in BIOPEP-UWM (http://www.uwm.edu.pl/biochemia/index.php/pl/biopep) (July 19, 2023) (Minkiewicz et al. 2019). For each protein, the frequency of release of fragments with ACE inhibitory activity by selected enzymes (A_E_), the relative frequency of release of fragments with ACE inhibitory activity by selected enzymes (W), and the theoretical degree of hydrolysis (DHt) were obtained by in silico hydrolysis. The most suitable protein hydrolysates and their associated enzymes were selected by comparing A_E_ and W values. By comparing the numbers of proteins suitable for digestion with each enzyme and the iBAQ values of these proteins, the optimal enzyme for in silico hydrolysis of WSPM was selected.

### In silico screening of ACE inhibitory peptides

WSPM was hydrolyzed in silico using the optimal enzyme, and the peptides obtained were further screened in silico. In the first instance, the potential bioactivities of the peptides were predicted using the server PeptideRanker (http://distilldeep.ucd.ie/PeptideRanker/) (July 20, 2022) (Mooney et al. 2012), and previously unreported peptides with a score ≥ 0.5 were selected for further analysis. The Innovagen tool (http://innovagen.com/proteomics-tools) (July 21, 2022) was then used to predict the water solubility of these peptides, and peptides with “Good water solubility” were selected for the prediction of ACE inhibitory activities. The A value obtained from the “calculations” tab in BIOPEP-UWM program, which represents the frequency of fragments with a certain bioactivity (chosen from a toolbar) in a protein sequence or a specific peptide, was then used to predict the potential ACE inhibitory activities of the peptides. In addition, the ADMET properties (https://admet.scbdd.com/calcpre/index/) (Dong et al. 2018), including HIA (human intestinal absorption) and BBB (blood–brain barrier) properties, were also evaluated (July 22, 2023). Finally, all potentially bioactive peptides exhibiting HIA^+^ and BBB^+^ properties were screened for toxicity using the ToxinPred tool (https://webs.iiitd.edu.in/raghava/toxinpred/design.php) (July 22, 2023) (Gupta et al. 2013).

### In silico analysis of the stability of ACE inhibitory peptides after the gastrointestinal (GI) digestion

To evaluate the stability of the ACE inhibitory peptides in vivo, the “enzyme action” tool in BIOPEP-UWM (http://www.uwm.edu.pl/biochemia/index.php/pl/biopep) (July 19, 2023) was applied again to predict the potential cleavage sites of the pepsin (pH 1.3), trypsin and chymotrypsin (A and C).

### Peptide synthesis

Novel non-toxic peptides exhibiting a PeptideRanker score > 0.5, good water solubility, potential ACE inhibitory activity, and acceptable ADMET properties were chosen for in vitro evaluation of ACE inhibitory activity. The retrieved sequences were employed to synthesize peptides with a purity of 98% using the solid-phase synthesis procedure (GL Biochem Co., Ltd, Shanghai, China). Finally, the purity and the amino acid sequences of the synthesized peptides were determined by HPLC (Chuangxintongheng Science and Technology Co., Ltd, Beijing, China) and LC–MS (Shimadzu LCMS-2020, Kyoto, Japan), respectively.

In vitro assay of ACE inhibitory activities of the selected peptides ACE inhibitory activity was evaluated in vitro according to previous methods with some modifications (Liao et al. 2018; Xie et al. 2022). All of the assays were carried out in 0.1 mol/L sodium borate buffer (containing 0.3 mol/L NaCl at pH 8.3). For each peptide of interest, 40 µL ACE solution (100 mU/mL) was pre-incubated with the peptide (60 µL) at 37 °C for 10 min. Next, 40 µL HHL solution (5.40 mmol/L) was added to the solution at 37 °C, and the reaction was allowed to proceed for 15 min at 37 °C. Finally, 60 µL 1 mol/L HCl was added to terminate the reaction. A borate buffer only control processed using the above procedure was also included for use as a blank. To quantify the amount of HA (hippuric acid) generated, 20 µL of reaction solution (pre-filtered using a 0.45 µm microporous membrane) was analyzed by HPLC (Waters e2695, Milford, USA) on a C18 column (150 × 4.6 mm, 5 µm; ThermoFisher Scientific, USA). The run conditions were as follows: mobile phase, methanol/water/phosphoric acid solution (15:84.9:0.1, V/V/V); flow rate, 1.0 mL/min; UV detection wavelength, 228 nm. All experiments were conducted in triplicate. ACE inhibitory activity was calculated according to the following equation:

1 $${\text{ACE inhibitory activity\% }} = \frac{{{\text{B}}{-}{\text{A}}}}{{\text{B}}} \times 100{\text{\% }}_{{}}$$

where A represents the peak area of HA in the sample, and B represents the peak area of HA in the blank.

### Determination of ACE inhibition kinetics

To elucidate the ACE inhibition kinetics of peptides of interest, different concentrations of HHL (0.27 mM, 0.54 mM, 1.08 mM, and 2.16 mM) and different concentrations of peptide (0 µM, 69 µM, 138 µM) were prepared in sodium borate buffer. The ACE activities of these test solutions were then determined. A Lineweaver–Burk plot was applied to determine the inhibition mode. The maximum enzyme reaction rate (*V*_max_) and Michaelis–Menten constant (*K*_m_) were calculated from these graphs. All experiments were repeated in triplicate.

### Molecular docking

The crystal structures of human ACE bound with lisinopril (ID: 1O86 and 2C6N) (Qi et al. 2018) were retrieved from the RCSB Protein Data Bank (https://www.rcsb.org) (July 25, 2023) (Burley et al. 2024). While 1O86 contains the coordinates for the testis ACE molecule (testis ACE is identical to the C-domain of somatic ACE), and 2C6N contains the coordinates for only the N domain of ACE. All heteroatoms, water molecules, and lisinopril were removed (with the exception of zinc and chloride ions), and hydrogen atoms were added. The 3D structure of the peptide has been depicted by ChemBio3D Ultra 14.0 (Cambridge Soft, USA) and the energy minimization was performed by using MMFF94 force field.

The structures of C-ACE, N-ACE, and peptide were converted into PDBQT format using AutodockTools-1.5.6. AutoDock Vina (Trott and Olson 2010) was then used for the molecular docking procedure. The docking center of 1O86 was set at (x = 40.582, y = 37.177, z = 43.444), and the grid box size (spacing) was 0.592. The docking center of 2C6N was set at (x = − 21.544, y = − 21.229, z = − 63.629), and the grid box size (spacing) was 0.913. The exhaustiveness value was 20. Finally, PyMOL (DeLano 2002) was used to analyze interactions between the peptide and ACE.

### Statistical analysis

All experiments were performed in triplicate, and the data are reported as mean ± standard deviation (SD). SPSS 20.0 software (SPSS Inc., Chicago IL, USA) and GraphPad Prism 9.0 (GraphPad Software Inc., San Diego, CA) were used for the statistical analysis of the data. Comparisons with a p < 0.05 were considered as statistically significant.

## Results and discussion

### Identification of proteins from WSPM

Shotgun proteomics is a useful tool for the rapid and direct analysis of complex protein mixtures (Wu and Maccoss 2002). Moreover, this technique is capable of generating whole profiles for these proteins. In the egg of *Pelodiscus sinensis*, proteins belonging to nine protein families have been identified (Qiu et al. 2021). In the present study, we report a shotgun proteomics analysis of WSPM. To our knowledge, this is the first time shotgun proteomics has been applied to these or similar samples. As presented in Table 1, a total of 39 proteins were identified from WSPM using this approach. The identified proteins belong (in the main) to the following protein families: immunoregulation-related protein (phosphopyruvate hydratase, SH3 domain-binding glutamic acid-rich protein, inducible heat shock protein 70), malic enzyme, tropomyosin, myosin, antioxidant enzyme (Peroxiredoxin), actin, kinase, troponin, galectin, ATPase (Cation-transporting P-type ATPase C-terminal domain-containing protein), G-protein coupled receptors, glycolytic enzymes (Triosephosphate isomerase), calmodulin, parvalbumin, titin, vinculin, nucleolin, transferrin, nebulin, and myomesin.

**Table 1:** The identified proteins in WSPM

Identified protein	Accession number	Unique peptides	Sequence coverage (%)	Molecular weight (KDa)	Score	iBAQ intensity
Phosphopyruvate hydratase	K7F8U8	10	32.7	47.315	171.57	6.32E + 07
Malic enzyme	K7G193	2	4	60.77	2.4226	5.86E + 07
SH3 domain-binding glutamic acid-rich protein	K7GBB4	5	19.3	24.996	23.022	3.52E + 07
Tropomyosin 1	K7FG05	9	55.3	28.404	323.31	1.50E + 07
Myosin-1B	K7FJP0	8	33.6	223.02	323.31	1.49E + 07
Peroxiredoxin	A0A6B9RHT1	4	24.6	22.235	16.791	1.44E + 07
Beta-actin	K7F0D9	2	27.3	41.633	38.087	1.06E + 07
Creatine kinase	A0A0G3FEK6	15	42	43.101	191.95	8.65E + 06
Phosphopyruvate hydratase	K7FRY2	5	25.8	47.296	119.82	7.42E + 06
Actin alpha 1, skeletal muscle	K7F4I9	3	31.3	42.051	109.9	6.25E + 06
Troponin I2, fast skeletal type	K7G5E5	5	30.7	20.732	56.222	5.41E + 06
Galectin	K7FWV6	3	20	14.906	1.4073	4.87E + 06
Inducible heat shock protein 70	F5CI36	5	17.2	69.906	117.9	3.22E + 06
Uncharacterized protein	K7FM18	1	10.2	9.1524	248.35	2.90E + 06
Cation-transporting P-type ATPase C-terminal domain-containing protein	K7GHR1	2	14.1	27.797	3.2772	2.84E + 06
Triosephosphate isomerase	K7G406	4	19.4	26.766	112.35	2.53E + 06
Mimecan	K7G6Z8	3	12.7	26.712	4.8477	2.16E + 06
G-protein coupled receptors family 1 profile domain-containing protein	K7FCP7	1	4.9	38.86	3.1967	2.12E + 06
Calmodulin 3	K7G387	4	30.4	16.706	17.114	1.62E + 06
Phosphoglycerate kinase	K7FQC8	6	24.5	44.738	76.585	1.49E + 06
Parvalbumin	K7G952	5	55	11.7	323.31	1.23E + 06
Myosin-1B-like	K7FM91	8	31.2	223.48	47.779	6.82E + 05
Catalase	J9QGW1	8	18.2	59.85	41.139	4.92E + 05
Glucose-6-phosphate isomerase	K7FXS1	9	21.8	54.641	54.512	4.23E + 05
Titin	K7G060	275	10.7	3881.7	323.31	3.79E + 05
Aldehyde dehydrogenase 9 family member A1	K7GI57	3	8.5	56.22	17.503	3.57E + 05
Alpha-1-antiproteinase-like	K7FR61	3	9.4	47.46	1.4206	2.98E + 05
Troponin I1, slow skeletal type	K7FJ27	3	17.6	21.867	30.198	2.73E + 05
Methanethiol oxidase	K7GJH4	2	4.2	53.083	6.2359	2.35E + 05
Four and a half LIM domains 1	K7FRC8	6	21.9	33.789	6.0207	2.00E + 05
Vinculin	K7FE14	15	18.8	118.53	132.68	1.71E + 05
Nucleolin	K7F2Y4	1	1.3	77.295	5.934	1.09E + 05
AHNAK nucleoprotein	K7FBT8	4	47.4	586.41	4.5687	9.20E + 04
Alpha fetoprotein	K7FQM8	23	2.9	69.527	323.31	7.17E + 04
Transferrin	K7FMT3	21	28.2	76.024	267.13	6.94E + 04
Talin 2	K7FQM1	2	27.8	271.75	50.782	5.48E + 04
WD repeat domain 33	K7F4M2	1	1.2	144.53	3.4022	3.74E + 04
Nebulin	K7G2Y3	22	4.2	768.64	104.15	3.26E + 04
Myomesin 2	K7GHV4	32	26.6	165.6	247.02	2.44E + 04

### In silico hydrolysis of WSPM

The peptides released from parent proteins typically exhibit different activities depending on the enzymes used (Carrera et al. 2020). In virtual enzyme digestions, poorly-performing enzymes can be rejected from a screening based on the activity of the hydrolysate or on DHt (Rawendra et al. 2013; Sun et al. 2022). However, this process is time-consuming and essentially blinded. As a consequence, in silico enzyme digestion is now more and more applied to known proteins to eliminate guesswork and to predict the release of peptides accurately and rapidly (Hakimi et al. 2022). In silico digestion of the given proteins by the specific protease, like pepsin and trypsin, is the usual workflow (Arámburo-Gálvez et al. 2022; Carrera et al. 2020; Chen et al. 2022; Hakimi et al. 2022; Kartal et al. 2020). However, there has been no research correlated with the selection of the optimal protease in the simulated digestion.

In the present study, the 39 WSPM proteins identified using shotgun proteomics were each digested in silico with each one of the six common proteases (chymotrypsin, trypsin, pepsin, papain, bromelain, and alkalase). The most suitable protease for each protein was then determined by comparing A_E_ and W values (see Table S1). The higher the A_E_ and W values, the higher the activity of the in silico digestion products, and the more suitable the protease for that protein. Using this procedure, the optimal protease for each protein was selected (selections marked in red in Table S1). For one previously uncharacterized protein (Protein Accession: K7G5E5), the A_E_ and W values were identical when papain and bromelain were used for the in silico digestion. However, the DHt values for these enzymes were observed to be different. As discussed above, DHt reflects the efficiency with which the enzyme produces peptides from the protein, and the higher the DHt value, the broader the specificity of the protease (Iwaniak et al. 2020). On this basis, bromelain (which exhibited a higher DHt value) was chosen as the optimal protease for the uncharacterized protein (Protein accession: K7G5E5). It should also be noted that in silico digestion is not possible for proteins like titin (Protein accession: K7G060), because the length (34,915 amino acids residues) of the protein exceeds the capacity limit for the server.

The total number of proteins most suitable for in silico digestion by each protease and the iBAQ values of their respective proteins were calculated and are listed in Table 2. Papain was suitable for the in silico digestion of 13 proteins, with iBAQ values covering 48.33% of the whole protein. Bromelain was the optimal enzyme for 25 proteins, with iBAQ values covering 51.53% of the whole protein. The iBAQ value of titin, which could not be digested using in silico methods, accounted for only 0.14% of the whole protein. The above results suggest that papain and bromelain can both be considered suitable for the virtual digestion of WSPM, which is partly in agreement with our actual protease screen (Sun et al. 2022).

**Table 2:** The number and the iBAQ values of proteins most suitable for each protease

Enzyme	Number of proteins	iBAQ values	iBAQ values coverage (%)
Papain	13	1.30E + 08	48.33
Bromelain	25	1.39E + 08	51.53
Total	38	2.69E + 08	99.86

### In silico screening of ACE inhibitory peptides from WSPM

The virtual digestions were performed on BIOPEP-UWM using two selected proteases: papain (EC 3.4.22.32), which mainly hydrolyzes the peptide bonds that follow Arg, Lys, Gly, and Phe residues (Liao et al. 2018; Qiu et al. 2021), and bromelain (EC 3.4.22.32), which specifically hydrolyzes the peptide bonds that follow Lys, Arg, Phe, and Tyr (Chen et al. 2022; Gajanan et al. 2016).

In total, 5670 peptides were released during the in silico digestion of 38 proteins by papain (Table S2); K7G2Y3 exhibited the richest abundance of fragments (894), while K7FM18 exhibited the fewest (22). All the released peptides were subsequently screened using the PeptideRanker program, yielding 1217 peptides with scores > 0.5. After secondary screening for the identification and removal of repetitive peptides and previously reported peptides, 663 peptides were evaluated for water solubility using the online Innovagen tool. In total, 318 peptides exhibited good water solubility, and these were evaluated in silico for ACE inhibitory activity. Analysis using the BIOPEP-UWM program identified 263 peptides that exhibited an A value indicative of ACE inhibitory activity. The ADMET properties, including BBB and HIA, of these 263 peptides were subsequently evaluated using ADMETlab. Only peptide IEWEF exhibited both BBB^+^ and HIA^+^ properties which was from myosin-1B-like. A subsequent evaluation of toxicity revealed that this peptide was “Non-toxic’’.

In parallel, we performed an in silico digestion of proteins by bromelain. In total, 8508 peptides were released during the in silico digestion of 38 proteins by bromelain (Table S3); K7G2Y3 exhibited the richest abundance of fragments (1633), while K7FM18 exhibited the fewest (22). Subsequent screening using the PeptideRanker program yielded 1710 peptides with scores > 0.5. After secondary screening, 522 unique, unknown peptides were further evaluated for water solubility using the online Innovagen tool. In total, 268 peptides exhibited good water solubility, and these were evaluated in silico for ACE inhibitory activity. Analysis using the BIOPEP-UWM program identified 218 peptides that exhibited an A value indicative of ACE inhibitory activity. Again, after the prediction of ADMET properties, IEWEF was the only satisfactory peptide identified and it was from myosin-1B.

Although in silico digestion of WSPM by papain and bromelain released an abundance of peptides, only one peptide (IEWEF) met all of the necessary criteria. To facilitate in vitro testing, peptide IEWEF was synthesized using the solid-phase procedure, purified by HPLC, and subsequently verified by LC–MS. The HPLC and LC–MS chromatogram of IEWEF is shown in Fig. S1. The purity of the synthesized peptide was 99.26%.

### Prediction of the stability of ACE inhibitory peptides after the GI digestion

The peptide IEWEF would be cutted into IEW and EF by chymotrypsin A and cutted into IE, W, E, and F by chymotrypsin C. The peptide IEWEF was stable in the stomach, and was mainly digested in the intestinal tract. The potential products IEW, EF, and IE were all identified ACE inhibitory peptides possessing weaker activities (van Platerink et al. 2008; Jimsheena and Gowda 2010; Li and Aluko 2010). Therefore, it could be speculated that the peptide IEWEF was not stable and may be digested into the products with decreased ACE inhibitory activities. The further in vivo studies of the stability of the peptide is still indispensable to verify the speculation.

### The in vitro ACE inhibitory activities of IEWEF

The IC_50_ value of peptide IEWEF against ACE was 41.33 ± 8.88 µM (Table S4). This is within the range (0.3 ~ 1000.0 µM) known to reduce blood pressure (Chen et al. 2022). There are several reported ACE inhibitory peptides similar to IEWEF in their amino acid compositions, including IE, EW, IEW, EF, LEF and IEEAF. The peptides IE and EW are both ACE inhibitory peptides isolated from milk hydrolysates, although their IC_50_ values have not been reported thus far (van Platerink et al. 2008). The peptide IEW from Arachin protein exhibits an IC_50_ value of 104 ± 3.0 µM (Jimsheena and Gowda 2010). The peptide EF is an ACE inhibitory peptide from pea protein with an IC_50_ value of 2980 ± 1240 µM (Li and Aluko 2010). The peptide LEF containing the same C-terminal is also an ACE inhibitory peptide from soybean and the IC_50_ value was 655.2 µM (Gu and Wu 2013). Meanwhile, the peptide IEEAF possessing the similar N and C-terminal showed around 40% inhibitory activity against ACE at 0.5 mg/mL (Girgih et al. 2014). Compared with these peptides, our novel peptide IEWEF exhibits stronger ACE inhibitory activity.

An analysis of the structure–activity relationship between peptide amino acid composition and ACE inhibitory activities revealed that the content of hydrophobic amino acids residues was proportional to ACE inhibitory activity (Ding et al. 2021). The presence of terminal hydrophobic amino acid residues played an especially important role in ACE inhibitory activities (Ding et al. 2021). The content of hydrophobic amino acids residues in peptide IEWEF was 60%, and the N-terminal and C-terminal amino acids residues were both hydrophobic.

Together, our results indicate that the novel peptide IEWEF exhibits strong ACE inhibitory activity. In addition, these results demonstrate that a workflow combing Shotgun analysis, in silico digestion and screening, and in vitro activity verification is a viable and effective strategy for the screening of novel ACE inhibitory peptides from a complex mixture of proteins.

### The Inhibition kinetics of IEWEF

**Fig. 1:**
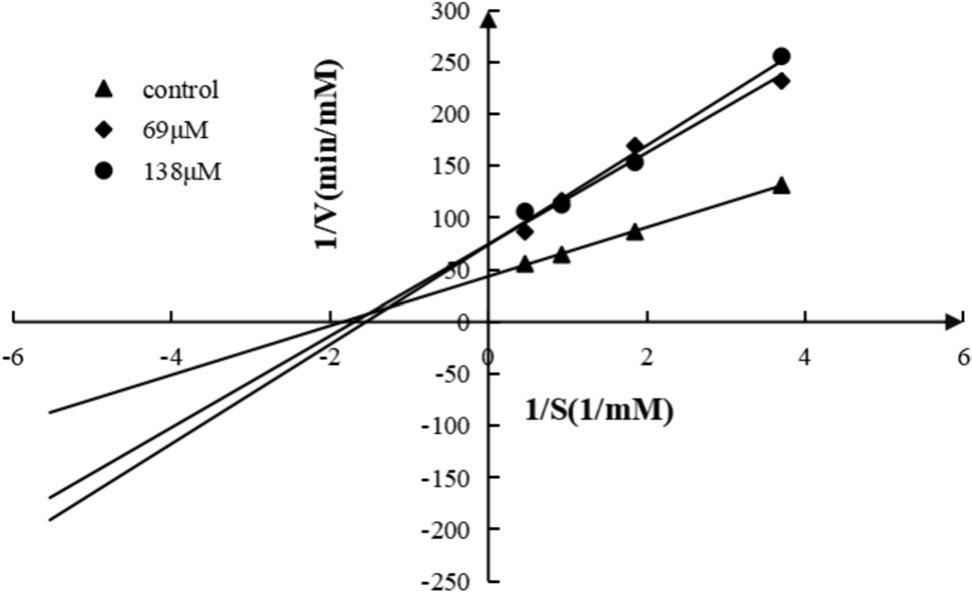
The Lineweaver–Burk plot of IEWEF at different concentrations, [1/V] and [1/S] represented the reciprocal of the reaction velocity and substrate concentration, respectively

The kinetics of IEWEF inhibition of ACE were determined by Lineweaver–Burk plot (Fig. 1). At different concentrations of IEWEF (0 µM, 69 µM, and 138 µM), the *K*_m_ remained constant, while *V*_max_ decreased significantly, indicating the characteristics of a non-competitive inhibitor (Table S5). We speculate that IEWEF interacts with the non-active domain of ACE to form an enzyme-inhibitor complex even in the presence of substrate (Xie et al. 2022). A number of non-competitive ACE inhibitory peptides have been reported in recent years, including GVSLPEW, GYGGVSLPEW, and VGINYW from *α*-lactalbumin (Wei et al. 2022), WGAP from rabbit meat (Chen et al. 2021), LLYQEPLGPVR from casein hydrolysate (Liu et al. 2020), KIGSRSRFEVT from the shiitake mushroom (Paisansak et al. 2021), KRER and KHMFK from *Carapax Trionycis* (the Shell of the Turtle *Pelodiscus sinensis*) (Liao et al. 2018), and PPLLFAAL from *Takifugu flavidus* (Su et al. 2021).

### Molecular docking analysis of IEWEF interaction with ACE

The molecular docking method is an important tool for exploring the interactions between peptides and ACE. ACE has two known isoforms, testis ACE (tACE) and somatic ACE (sACE). sACE is composed of a N-terminal domain and a C-terminal domain (N-ACE and C-ACE) (Qi et al. 2018). While the two domains share 65% sequence similarity, they exhibit marked differences in their specificities for substrate, inhibitor, and chloride ions (Song et al. 2021). Thus, N-ACE hydrolyzes the anti-inflammatory peptide N-acetyl-SDKP, C-ACE is mainly responsible for the hydrolysis of angiotensin I, and bradykinin is hydrolyzed by both domains (Alfaro et al. 2020). With these characteristics in mind, domain selectivity has been the emphasis during the development of a new generation of ACE inhibitors. Song et al. studied the selective inhibition of several tyrosine-containing dipeptides by molecular docking, and the results achieved were consistent with those obtained from in vitro experiments (Song et al. 2021).

**Fig. 2:**
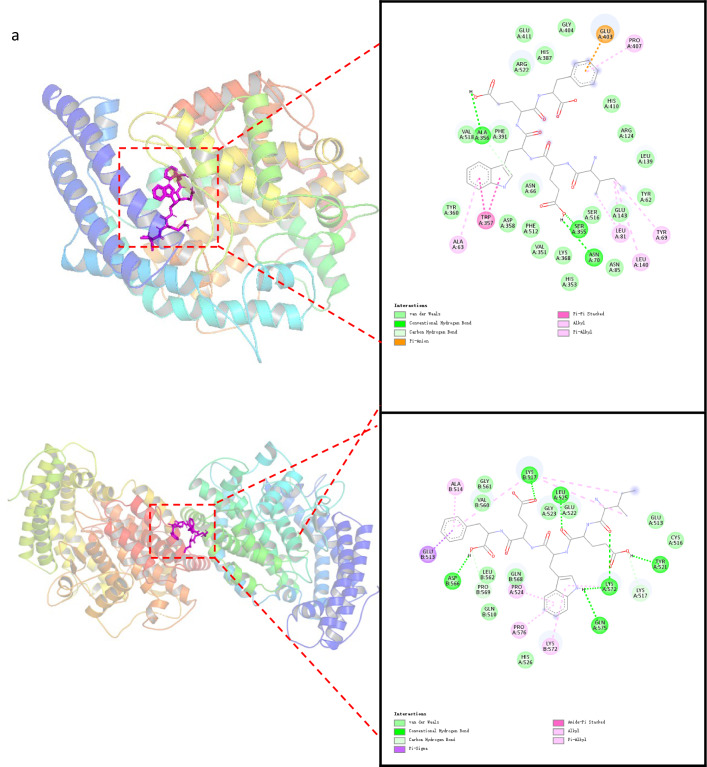
3D and 2D models of docked poses and interaction of IEWEF with C-ACE (**a**) and N-ACE (**b**)

The interactions between peptide IEWEF and C/N-ACE are illustrated in Fig. 2. Multiple different kinds of interactions have been observed between active peptides and ACE, including hydrogen bonds, hydrophobic interactions, van der Waals’s force, π bonds, and electrostatic forces (Wei et al. 2022; Kheeree et al. 2020).

As shown in Fig. 2a, the peptide IEWEF bound to C-ACE with three hydrogen bonds between Asn70, Ser355, Ala356 and the hydrogen atom on carboxyl group of IEWEF. An untypical π–π stacking interaction was generated between the indole portion of IEWEF and residues of Trp357. This is due to the proximity of the hydrophobic residues of C-ACE to the indole ring of IEWEF. The Van der Waals interactive forces were formed by the residues of Asn66, His353, Asp358, Val351, Lys368, Phe512, Ser516, Asn85, Glu143, Tyr62, Leu139, Arg124, Phe391, Gly404, His410, His387, Arg522, Glu411, Val518 and Tyr360 of C-ACE with IEWEF. There is also a π-anion interaction between Glu403 of C-ACE and IEWEF, because of the interaction between anions and electron deficient aromatic ring centers which was advantageous in terms of energy.

As shown in Fig. 2b, seven hydrogen bonds were formed between Gln575, Lys572, Tyr521, Leu525, Lys517, Asp566 of N-ACE and the hydrogen atom on carboxyl group and amino, carbonyl of IEWEF, respectively. Untypical π–δ and π-amide stacking interaction was created between the benzene ring of IEWEF and the residue of Glu513 and Ala514. π-alkyl or alkyl stacking interaction was also presented between the benzene ring and indole ring of IEWEF and the residue of Pro524, Pro576 and Lys572. The Van der Waals interactive forces were formed by the residues of His526, Cys516, Lys517, Glu513, Gly561, Val560, Leu562, Pro569, Gln510, Gln568 and Gly523 of N-ACE with IEWEF.

The peptide IEWEF interacted with only two residues (His353 and His387) in the active site of C-ACE, which indicated that the peptide mainly coordinated with the non-active domain of ACE (Duan et al. 2023).

## Conclusion

In the present paper, 39 proteins in *Pelodiscus sinensis* meat were identified by Shotgun analysis for the first time. In addition, an abundance of peptides released from these proteins by virtual digestion were identified and screened. By combining a series of virtual digestion and screening procedures, an efficient in silico screening platform for ACE inhibitory peptides was established. Using this platform, a novel ACE inhibitory peptide IEWEF (Ile-Glu-Trp-Glu-Phe) with an IC_50_ value of 41.33 µM was successfully screened out. An in vitro enzyme inhibition kinetics analysis of the synthesized peptide and a molecular docking study provide evidence that peptide IEWEF is a non-competitive ACE inhibitor, and that it mainly binds to ACE outside of the active pockets.

Our findings provide additional evidence for the presence of ACE inhibitory peptides in WSPM. In addition, they demonstrate that a new workflow established in this study can be applied for the rapid and accurate identification of novel ACE inhibitory peptides from complex protein mixtures. Future research should include a clarification of the domain selectivity of IEWEF, an investigation of the in vivo anti-ACE activities of IEWEF, and a search for more novel ACE inhibitory peptides using the established workflow on other protein mixtures.

## Supplementary Information

Below is the link to the electronic supplementary material.

Supplementary file1 (DOCX 306 KB)

## Data Availability

All data included in this study are available upon request by contact with the first authors and the corresponding authors.
