# Supplementary material for: A novel ACE inhibitory peptide from Pelodiscus sinensis Wiegmann meat water-soluble protein hydrolysate
Source: Amino Acids. 2024 Jun 7;56(1):40. doi: 10.1007/s00726-024-03399-1 (PMC11585513; doi:10.1007/s00726-024-03399-1)
Supplement: Supplementary file 1 — Supplementary file1 (DOCX 306 KB) [file 726_2024_3399_MOESM1_ESM.docx]

**A novel ACE inhibitory peptide from *Pelodiscus sinensis* Wiegmann meat water-soluble protein hydrolysate**

Pengying Liao^1,2,4#^, Huayu Liu^1#^, Xueqin Sun^1^, Xinrui Zhang^1^, Miao Zhang^4^, Xianyou Wang^3 *^, Jun Chen^5*^

^1^ College of Pharmacy, Guangxi University of Chinese Medicine, Nanning 530200, Guangxi, China

^2^ Guangxi Key Laboratory of Efficacy Study on Chinese Materia Medica, Guangxi University of Chinese Medicine, Nanning 530200, Guangxi, China

^3^ School of Pharmacy, Henan University, Kaifeng 475004, Henan, China

^4^ Guangxi Key Laboratory of Zhuang and Yao Ethnic Medicine, Guangxi University of Chinese Medicine, Nanning 530200, Guangxi, China

*^5^* Teaching Experiment and Training Centre, Guangxi University of Chinese Medicine, Nanning 530200, China

Pengying Liao: [gxlpy@163.com](mailto:gxlpy@163.com) (ORCID: 0009−0004−6697−1990)

* Corresponding authors.

Xianyou Wang: w_xianyou@163.com

Jun Chen : 543767193@qq.com

^#^Pengying Liao and Huayu Liu have contributed equally to this work.

Table S1 Values of the BIOPEP-UWM parameters of the simulated digestion of 38 proteins by six different proteases

| Protein Accession | protease | DHt [%] | A_E_ | W |
| --- | --- | --- | --- | --- |
| K7F8U8 | C^1^ | 22.87 | 0.0157 | 0.0336 |
|  | T | 11.50 | 0.0068 | 0.0172 |
|  | Pep | 12.66 | 0.0100 | 0.0253 |
|  | Pa | 35.98 | 0.0504 | 0.1273 |
|  | B | 51.78 | 0.0572 | 0.1445 |
|  | A | 31.99 | 0.0315 | 0.0796 |
| K7G193 | C | 26.74 | 0.0391 | 0.0862 |
|  | T | 10.16 | 0.0107 | 0.0236 |
|  | Pep | 14.44 | 0.0053 | 0.0117 |
|  | Pa | 39.93 | 0.0605 | 0.1333 |
|  | B | 50.09 | 0.0587 | 0.129 |
|  | A | 28.34 | 0.0267 | 0.0588 |
| K7GBB4 | C | 20.61 | 0.0175 | 0.0445 |
|  | T | 9.65 | 0 | 0 |
|  | Pep | 9.65 | 0.0087 | 0.022 |
|  | Pa | 31.58 | 0.0306 | 0.0779 |
|  | B | 42.54 | 0.0306 | 0.0779 |
|  | A | 26.18 | 0.0246 | 0.0527 |
| K7FG05 | C | 19.76 | 0 | 0 |
|  | T | 15.57 | 0.0060 | 0.0202 |
|  | Pep | 10.18 | 0.0060 | 0.0202 |
|  | Pa | 33.20 | 0.0472 | 0.1378 |
|  | B | 41.11 | 0.0433 | 0.1264 |
|  | A | 20.95 | - | - |
| K7FJP0 | C | 23.82 | 0.0165 | 0.0426 |
|  | T | 15.72 | 0.0150 | 0.0387 |
|  | Pep | 12.71 | 0.0035 | 0.0090 |
|  | Pa | 36.79 | 0.0400 | 0.1033 |
|  | B | 43.69 | 0.0550 | 0.1420 |
|  | A | 23.92 | 0.0150 | 0.0387 |

| A0A6B9RHT1 | C | 23.53 | 0.0341 | 0.0768 |
| --- | --- | --- | --- | --- |
|  | T | 11.76 | 0.0049 | 0.0110 |
|  | Pep | 13.24 | 0.0146 | 0.0329 |
|  | Pa | 35.78 | 0.0439 | 0.0989 |
|  | B | 48.04 | 0.0390 | 0.0879 |
|  | A | 26.96 | 0.0341 | 0.0768 |
| K7F0D9 | C | 23.64 | 0.0207 | 0.0392 |
|  | T | 9.61 | 0.0155 | 0.0293 |
|  | Pep | 9.87 | 0.0078 | 0.0148 |
|  | Pa | 35.84 | 0.0440 | 0.0833 |
|  | B | 48.31 | 0.0544 | 0.1029 |
|  | A | 25.71 | 0.0207 | 0.0392 |
| A0A0G3FEK6 | C | 28.57 | 0.0204 | 0.0438 |
|  | T | 13.01 | 0.0153 | 0.0329 |
|  | Pep | 13.78 | 0.0076 | 0.0163 |
|  | Pa | 36.73 | 0.0356 | 0.0765 |
|  | B | 46.94 | 0.0433 | 0.0930 |
|  | A | 27.30 | 0.0331 | 0.0711 |
| K7FRY2 | C | 23.71 | 0.0134 | 0.0314 |
|  | T | 12.30 | 0.0089 | 0.0209 |
|  | Pep | 11.63 | 0.0022 | 0.0052 |
|  | Pa | 34.90 | 0.0402 | 0.0943 |
|  | B | 50.56 | 0.0580 | 0.1361 |
|  | A | 27.52 | 0.0246 | 0.0577 |
| K7F4I9 | C | 24.48 | 0.0231 | 0.0434 |
|  | T | 9.54 | 0.0154 | 0.0289 |
|  | Pep | 9.54 | 0.0077 | 0.0145 |
|  | Pa | 35.05 | 0.0540 | 0.1015 |
|  | B | 47.16 | 0.0566 | 0.1064 |
|  | A | 24.23 | 0.0231 | 0.0434 |

| K7G5E5 | C | 23.08 | - | - |
| --- | --- | --- | --- | --- |
|  | T | 21.98 | 0.0219 | 0.0466 |
|  | Pep | 12.09 | - | - |
|  | Pa | 25.71 | 0.0437 | 0.0930 |
|  | B | 45.05 | 0.0437 | 0.0930 |
|  | A | 21.43 | 0.0109 | 0.0232 |
| K7FWV6 | C | 27.54 | 0.0288 | 0.0755 |
|  | T | 10.87 | - | - |
|  | Pep | 13.77 | 0.0144 | 0.0378 |
|  | Pa | 36.96 | 0.0576 | 0.1511 |
|  | B | 50.72 | 0.0647 | 0.1697 |
|  | A | 27.54 | 0.0288 | 0.0755 |
| F5CI36 | C | 20.80 | 0.0046 | 0.0103 |
|  | T | 12.84 | 0.0122 | 0.0273 |
|  | Pep | 10.09 | 0.0015 | 0.0034 |
|  | Pa | 39.14 | 0.0611 | 0.1366 |
|  | B | 49.39 | 0.0641 | 0.1433 |
|  | A | 23.70 | 0.0122 | 0.0273 |
| K7FM18 | C | 20.22 | 0.0222 | 0.0434 |
|  | T | 10.11 | - | - |
|  | Pep | 7.87 | - | - |
|  | Pa | 42.70 | 0.0444 | 0.0869 |
|  | B | 52.81 | 0.0889 | 0.1739 |
|  | A | 19.10 | 0.0333 | 0.0652 |
| K7GHR1 | C | 21.76 | 0.0152 | 0.0320 |
|  | T | 10.31 | - | - |
|  | Pep | 12.21 | 0.0076 | 0.0160 |
|  | Pa | 38.17 | 0.0722 | 0.1519 |
|  | B | 51.15 | 0.0760 | 0.1599 |
|  | A | 25.57 | 0.0304 | 0.0640 |
| K7G406 | C | 20.00 | 0.0156 | 0.0353 |
|  | T | 11.37 | 0.0078 | 0.0177 |
|  | Pep | 9.80 | 0.0039 | 0.0088 |
|  | Pa | 36.86 | 0.0469 | 0.1063 |
|  | B | 51.37 | 0.0703 | 0.1593 |
|  | A | 25.10 | 0.0273 | 0.0618 |
| K7G6Z8 | C | 30.17 | 0.0247 | 0.0588 |
|  | T | 11.57 | - | - |
|  | Pep | 16.53 | 0.0082 | 0.0195 |
|  | Pa | 34.71 | 0.0412 | 0.0981 |
|  | B | 47.52 | 0.0494 | 0.1177 |
|  | A | 28.93 | 0.0247 | 0.0588 |

| K7FCP7 | C | 34.08 | 0.0334 | 0.0784 |
| --- | --- | --- | --- | --- |
|  | T | 7.26 | 0.0056 | 0.0131 |
|  | Pep | 20.95 | 0.0195 | 0.0458 |
|  | Pa | 42.18 | 0.0724 | 0.1699 |
|  | B | 59.50 | 0.0557 | 0.1307 |
|  | A | 39.94 | 0.0613 | 0.1438 |
| K7G387 | C | 23.18 | 0.0132 | 0.0318 |
|  | T | 8.61 | - | - |
|  | Pep | 11.26 | - | - |
|  | Pa | 38.41 | 0.0724 | 0.1747 |
|  | B | 45.03 | 0.0592 | 0.1428 |
|  | A | 19.21 | 0.0132 | 0.0318 |
| K7FQC8 | C | 23.83 | 0.0117 | 0.0287 |
|  | T | 12.15 | 0.0047 | 0.0115 |
|  | Pep | 12.15 | 0.0047 | 0.0115 |
|  | Pa | 35.28 | 0.0443 | 0.1086 |
|  | B | 50.47 | 0.0466 | 0.1142 |
|  | A | 27.10 | 0.0326 | 0.0799 |
| K7G952 | C | 21.82 | - | - |
|  | T | 11.82 | 0.0090 | 0.0208 |
|  | Pep | 16.36 | - | - |
|  | Pa | 43.64 | 0.0721 | 0.1667 |
|  | B | 54.55 | 0.0631 | 0.1459 |
|  | A | 29.09 | 0.0180 | 0.0416 |

| K7FJ27 | C | 23.32 | 0.0155 | 0.0381 |
| --- | --- | --- | --- | --- |
|  | T | 22.80 | 0.0309 | 0.0759 |
|  | Pep | 11.92 | 0.0103 | 0.0253 |
|  | Pa | 35.23 | 0.0567 | 0.1392 |
|  | B | 47.15 | 0.0670 | 0.1645 |
|  | A | 24.87 | 0.0309 | 0.0759 |
| K7GJH4 | C | 28.04 | 0.0288 | 0.0651 |
|  | T | 10.51 | 0.0021 | 0.0047 |
|  | Pep | 11.34 | 0.0041 | 0.0093 |
|  | Pa | 33.40 | 0.0432 | 0.0976 |
|  | B | 45.36 | 0.0638 | 0.1442 |
|  | A | 28.87 | 0.0350 | 0.0791 |
| K7FRC8 | C | 23.36 | 0.0197 | 0.0532 |
|  | T | 14.7 | - | - |
|  | Pep | 9.54 | 0.0033 | 0.0089 |
|  | Pa | 30.92 | 0.0328 | 0.0885 |
|  | B | 42.10 | 0.0426 | 0.1150 |
|  | A | 25.99 | 0.0164 | 0.0443 |
| K7FE14 | C | 19.27 | 0.0045 | 0.0112 |
|  | T | 12.63 | 0.0072 | 0.0179 |
|  | Pep | 10.04 | 0.0018 | 0.0045 |
|  | Pa | 40.23 | 0.0555 | 0.1381 |
|  | B | 49.73 | 0.0501 | 0.1246 |
|  | A | 21.68 | 0.0081 | 0.0201 |
| K7F2Y4 | C | 13.92 | 0.0123 | 0.0262 |
|  | T | 15.83 | 0.0082 | 0.0175 |
|  | Pep | 7.78 | 0.0041 | 0.0087 |
|  | Pa | 36.43 | 0.0490 | 0.1045 |
|  | B | 47.61 | 0.0572 | 0.1220 |
|  | A | 20.05 | 0.0177 | 0.0378 |

| K7FM91 | C | 24.89 | 0.0174 | 0.0451 |
| --- | --- | --- | --- | --- |
|  | T | 15.41 | 0.0150 | 0.0389 |
|  | Pep | 12.97 | 0.0040 | 0.0104 |
|  | Pa | 38.86 | 0.0389 | 0.1008 |
|  | B | 44.24 | 0.0518 | 0.1343 |
|  | A | 24.94 | 0.0174 | 0.0451 |
| J9QGW1 | C | 27.26 | 0.0294 | 0.0681 |
|  | T | 11.42 | 0.0055 | 0.0127 |
|  | Pep | 11.05 | 0.0037 | 0.0086 |
|  | Pa | 35.91 | 0.0551 | 0.1275 |
|  | B | 46.22 | 0.0441 | 0.1021 |
|  | A | 26.52 | 0.0257 | 0.0595 |
| K7FXS1 | C | 29.31 | 0.0217 | 0.0492 |
|  | T | 10.30 | 0.0059 | 0.0134 |
|  | Pep | 13.47 | 0.0059 | 0.0134 |
|  | Pa | 38.22 | 0.0573 | 0.1300 |
|  | B | 49.11 | 0.0593 | 0.1346 |
|  | A | 27.92 | 0.0277 | 0.0629 |
| K7GI57 | C | 23.58 | 0.0169 | 0.0363 |
|  | T | 9.34 | 0.0019 | 0.0041 |
|  | Pep | 12.08 | 0.0056 | 0.0120 |
|  | Pa | 39.43 | 0.0584 | 0.1255 |
|  | B | 53.77 | 0.0490 | 0.1053 |
|  | A | 28.30 | 0.0339 | 0.0729 |
| K7FR61 | C | 23.01 | 0.0280 | 0.0751 |
|  | T | 10.05 | 0.0070 | 0.0188 |
|  | Pep | 15.89 | 0.0117 | 0.0314 |
|  | Pa | 34.35 | 0.1437 | 0.0002 |
|  | B | 46.26 | 0.1314 | 0.00021 |
|  | A | 28.27 | 0.0751 | 0.0092 |

| K7FBT8 | C | 17.93 | 0.0062 | 0.0141 |
| --- | --- | --- | --- | --- |
|  | T | 15.15 | 0.0037 | 0.0084 |
|  | Pep | 10.41 | 0.0034 | 0.0077 |
|  | Pa | 27.19 | 0.0604 | 0.1369 |
|  | B | 38.73 | 0.0704 | 0.1596 |
|  | A | 22.07 | 0.0092 | 0.0209 |
| K7FQM8 | C | 24.72 | 0.0206 | 0.0540 |
|  | T | 11.89 | 0.0063 | 0.0165 |
|  | Pep | 13.79 | 0.0079 | 0.0207 |
|  | Pa | 35.34 | 0.0396 | 0.1039 |
|  | B | 42.79 | 0.0491 | 0.1288 |
|  | A | 25.04 | 0.0190 | 0.0498 |
| K7FMT3 | C | 22.02 | 0.0112 | 0.0269 |
|  | T | 10.94 | 0.0070 | 0.0168 |
|  | Pep | 10.94 | 0.0028 | 0.0067 |
|  | Pa | 36.75 | 0.0448 | 0.1077 |
|  | B | 51.33 | 0.0392 | 0.0942 |
|  | A | 29.45 | 0.0182 | 0.0438 |
| K7FQM1 | C | 19.66 | 0.0107 | 0.0291 |
|  | T | 10.17 | 0.0061 | 0.0166 |
|  | Pep | 10.70 | 0.0034 | 0.0093 |
|  | Pa | 40.88 | 0.0427 | 0.1162 |
|  | B | 53.64 | 0.0465 | 0.1265 |
|  | A | 24.95 | 0.0137 | 0.0373 |
| K7F4M2 | C | 23.00 | 0.0205 | 0.0332 |
|  | T | 11.28 | 0.0102 | 0.0165 |
|  | Pep | 9.01 | 0.0029 | 0.0047 |
|  | Pa | 40.42 | 0.0864 | 0.1398 |
|  | B | 45.35 | 0.0578 | 0.0935 |
|  | A | 18.53 | 0.0110 | 0.0178 |
| K7G2Y3 | C | 26.75 | 0.0219 | 0.0561 |
|  | T | 15.38 | 0.0150 | 0.0384 |
|  | Pep | 8.94 | 0.0022 | 0.0056 |
|  | Pa | 30.20 | 0.0251 | 0.0643 |
|  | B | 40.22 | 0.0359 | 0.0920 |
|  | A | 27.34 | 0.0251 | 0.0643 |
| K7GHV4 | C | 24.37 | 0.0159 | 0.0402 |
|  | T | 11.59 | 0.0099 | 0.0251 |
|  | Pep | 10.46 | 0.0040 | 0.0101 |
|  | Pa | 33.91 | 0.0377 | 0.0954 |
|  | B | 48.15 | 0.0430 | 0.1088 |
|  | A | 29.47 | 0.0285 | 0.0721 |

^1^ C-chymotrypsin, T-trypsin, Pep-pepsin, Pa-papain, B-bromelain, A-alcalase

Table S2 The *in silico* screening results of ACE inhibitory peptides from the virtual digestion of WSPM by papain

| Accession number | The length of the amino acids residues | The number of the peptides released by *in silico* hydrolysis | The number of the peptides with Peptide ranker score > 0.5 | The number of the unreported peptides with Peptide ranker score > 0.5 without repetition | The number of the unreported peptides with good water-solubility and with Peptide ranker score > 0.5 and without repetition | The number of the unreported peptides with good water-solubility, potent ACE inhibitory activities and without repetition |
| --- | --- | --- | --- | --- | --- | --- |
| K7F8U8 | 434 | 105 | 21 | 10 | 4 | 3 |
| K7G193 | 544 | 122 | 29 | 16 | 2 | 2 |
| K7GBB4 | 223 | 50 | 11 | 8 | 3 | 3 |
| K7FG05 | 246 | 58 | 3 | 3 | 1 | 0 |
| K7FJP0 | 1935 | 361 | 60 | 37 | 14 | 9 |
| A0A6B9RHT1 | 199 | 47 | 11 | 6 | 2 | 1 |
| K7F0D9 | 374 | 84 | 19 | 13 | 4 | 3 |
| A0A0G3FEK6 | 381 | 81 | 17 | 10 | 5 | 3 |
| K7FRY2 | 434 | 103 | 19 | 2 | 2 | 2 |
| K7F4I9 | 377 | 81 | 18 | 2 | 1 | 1 |
| K7G5E5 | 179 | 41 | 8 | 6 | 4 | 2 |
| K7FWV6 | 135 | 37 | 10 | 3 | 2 | 1 |
| F5CI36 | 635 | 147 | 21 | 6 | 1 | 1 |
| K7FM18 | 88 | 22 | 8 | 4 | 6 | 5 |
| K7GHR1 | 255 | 65 | 23 | 10 | 2 | 2 |
| K7G406 | 248 | 58 | 13 | 6 | 1 | 1 |
| K7G6Z8 | 237 | 59 | 9 | 6 | 4 | 3 |
| K7FCP7 | 349 | 80 | 31 | 17 | 1 | 1 |
| K7G387 | 148 | 34 | 6 | 2 | 8 | 7 |
| K7FQC8 | 417 | 88 | 23 | 14 | 2 | 2 |
| K7G952 | 109 | 26 | 4 | 2 | 1 | 1 |
| K7FM91 | 1942 | 365 | 62 | 6 | 7 | 7 |
| J9QGW1 | 528 | 118 | 29 | 18 | 10 | 6 |
| K7FXS1 | 490 | 103 | 30 | 20 | 0 | 0 |
| K7GI57 | 515 | 108 | 33 | 14 | 6 | 4 |
| K7FR61 | 417 | 92 | 27 | 13 | 5 | 5 |
| K7FJ27 | 188 | 48 | 11 | 2 | 1 | 0 |
| K7GJH4 | 472 | 110 | 37 | 18 | 8 | 8 |
| K7FRC8 | 297 | 62 | 20 | 13 | 6 | 5 |
| K7FE14 | 1081 | 212 | 37 | 22 | 12 | 9 |
| K7F2Y4 | 712 | 129 | 20 | 8 | 6 | 4 |
| K7FBT8 | 5468 | 464 | 87 | 65 | 52 | 50 |
| K7FQM8 | 612 | 134 | 41 | 19 | 10 | 7 |
| K7FMT3 | 692 | 147 | 43 | 26 | 13 | 10 |
| K7FQM1 | 2542 | 418 | 60 | 30 | 14 | 10 |
| K7F4M2 | 1322 | 234 | 91 | 57 | 23 | 22 |
| K7G2Y3 | 6636 | 894 | 168 | 117 | 57 | 49 |
| K7GHV4 | 1463 | 283 | 57 | 32 | 18 | 14 |
| Total |  | 5670 | 1217 | 663 | 318 | 263 |

Table S3 The *in silico* screening results of ACE inhibitory peptides from the virtual digestion of WSPM by bromelain

| Accession number | The length of the amino acids residues | The number of the peptides released by *in silico* hydrolysis | The number of the peptides with Peptide ranker score > 0.5 | The number of the unreported peptides with Peptide ranker score > 0.5 without repetition | The number of the unreported peptides with good water-solubility and with Peptide ranker score > 0.5 and without repetition | The number of the unreported peptides with good water-solubility, potent ACE inhibitory activities and without repetition |
| --- | --- | --- | --- | --- | --- | --- |
| K7F8U8 | 434 | 112 | 27 | 10 | 7 | 5 |
| K7G193 | 544 | 130 | 23 | 8 | 1 | 1 |
| K7GBB4 | 223 | 46 | 10 | 7 | 3 | 3 |
| K7FG05 | 246 | 63 | 2 | 1 | 0 | 0 |
| K7FJP0 | 1935 | 492 | 67 | 35 | 12 | 9 |
| A0A6B9RHT1 | 199 | 51 | 16 | 7 | 2 | 2 |
| K7F0D9 | 374 | 89 | 16 | 7 | 3 | 2 |
| A0A0G3FEK6 | 381 | 84 | 18 | 9 | 4 | 4 |
| K7FRY2 | 434 | 115 | 26 | 1 | 1 | 1 |
| K7F4I9 | 377 | 88 | 16 | 2 | 1 | 0 |
| K7G5E5 | 179 | 45 | 9 | 4 | 4 | 3 |
| K7FWV6 | 135 | 40 | 9 | 3 | 2 | 1 |
| F5CI36 | 635 | 161 | 25 | 4 | 1 | 1 |
| K7FM18 | 88 | 22 | 8 | 3 | 0 | 0 |
| K7GHR1 | 255 | 66 | 24 | 10 | 5 | 4 |
| K7G406 | 248 | 65 | 14 | 3 | 2 | 2 |
| K7G6Z8 | 237 | 62 | 13 | 5 | 1 | 1 |
| K7FCP7 | 349 | 78 | 32 | 12 | 4 | 3 |
| K7G387 | 148 | 40 | 6 | 2 | 1 | 1 |
| K7FQC8 | 417 | 105 | 22 | 9 | 5 | 4 |
| K7G952 | 109 | 28 | 3 | 1 | 1 | 1 |
| K7FM91 | 1942 | 497 | 72 | 5 | 1 | 1 |
| J9QGW1 | 528 | 133 | 27 | 14 | 7 | 5 |
| K7FXS1 | 490 | 125 | 30 | 15 | 10 | 6 |
| K7GI57 | 515 | 128 | 37 | 10 | 3 | 0 |
| K7FR61 | 417 | 106 | 27 | 11 | 5 | 2 |
| K7FJ27 | 188 | 53 | 13 | 2 | 1 | 5 |
| K7GJH4 | 472 | 126 | 40 | 19 | 9 | 8 |
| K7FRC8 | 297 | 70 | 23 | 10 | 5 | 4 |
| K7FE14 | 1081 | 270 | 46 | 24 | 13 | 12 |
| K7F2Y4 | 712 | 162 | 29 | 7 | 5 | 3 |
| K7FBT8 | 5468 | 1599 | 305 | 44 | 37 | 35 |
| K7FQM8 | 612 | 147 | 41 | 17 | 12 | 7 |
| K7FMT3 | 692 | 171 | 46 | 22 | 12 | 10 |
| K7FQM1 | 2542 | 623 | 92 | 27 | 12 | 9 |
| K7F4M2 | 1322 | 326 | 147 | 52 | 23 | 20 |
| K7G2Y3 | 6636 | 1633 | 280 | 75 | 37 | 31 |
| K7GHV4 | 1463 | 357 | 69 | 25 | 16 | 12 |
| Total |  | 8508 | 1710 | 522 | 268 | 218 |

Table S4 The ACE inhibitory activities of IEWEF at different concentrations (*n* = 3)

| Concentrations (*μ*mol/L) | ACE inhibitory activities (%) | | |
| --- | --- | --- | --- |
|  | 1 | 2 | 3 |
| 86.57 | 58.37 | 57.79 | 57.92 |
| 173.13 | 65.33 | 59.34 | 64.99 |
| 346.26 | 72.22 | 69.33 | 70.50 |
| 692.52 | 66.83 | 74.43 | 74.54 |
| 2770.08 | 91.15 | 91.03 | 91.05 |

Table S5 The kinetic constant of ACE catalyzed reaction at different concentrations of IEWEF

| Catalytic constants | Control | IEWEF | |
| --- | --- | --- | --- |
| Concentration（*μ*mol/L） | 0.00 | 69.00 | 138.00 |
| *V*_max_ (*μ*mol/L·min) | 18.00 | 11.58 | 9.43 |
| *K*_m_ (mmol/L) | 0.54 | 0.59 | 0.65 |


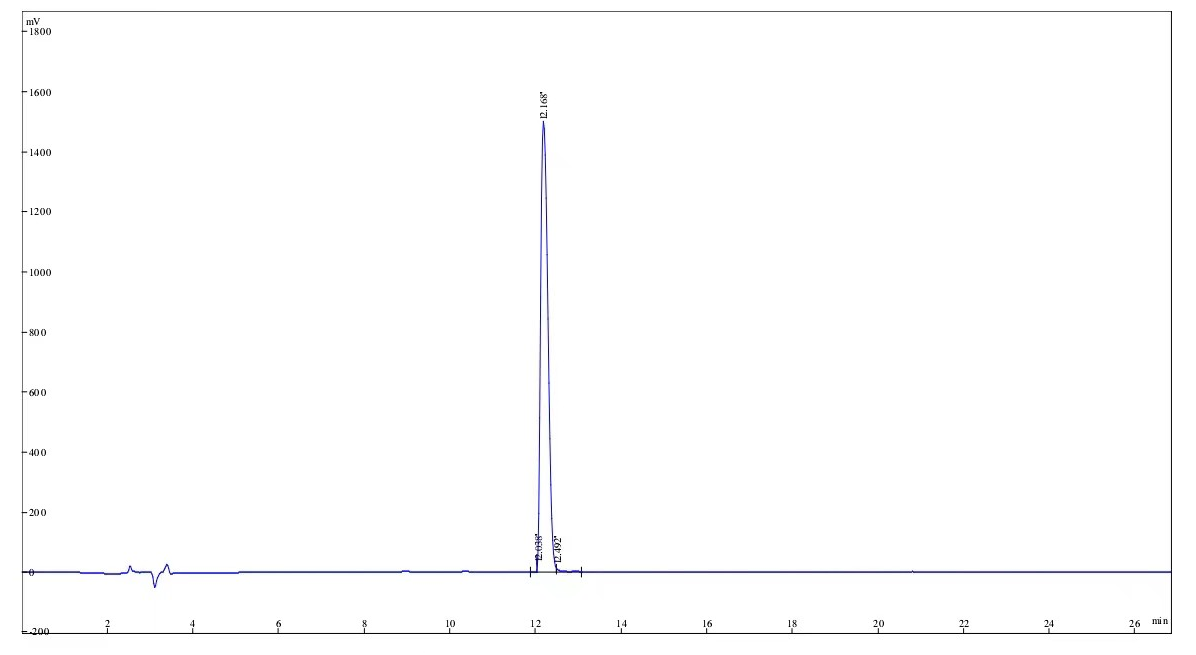


A
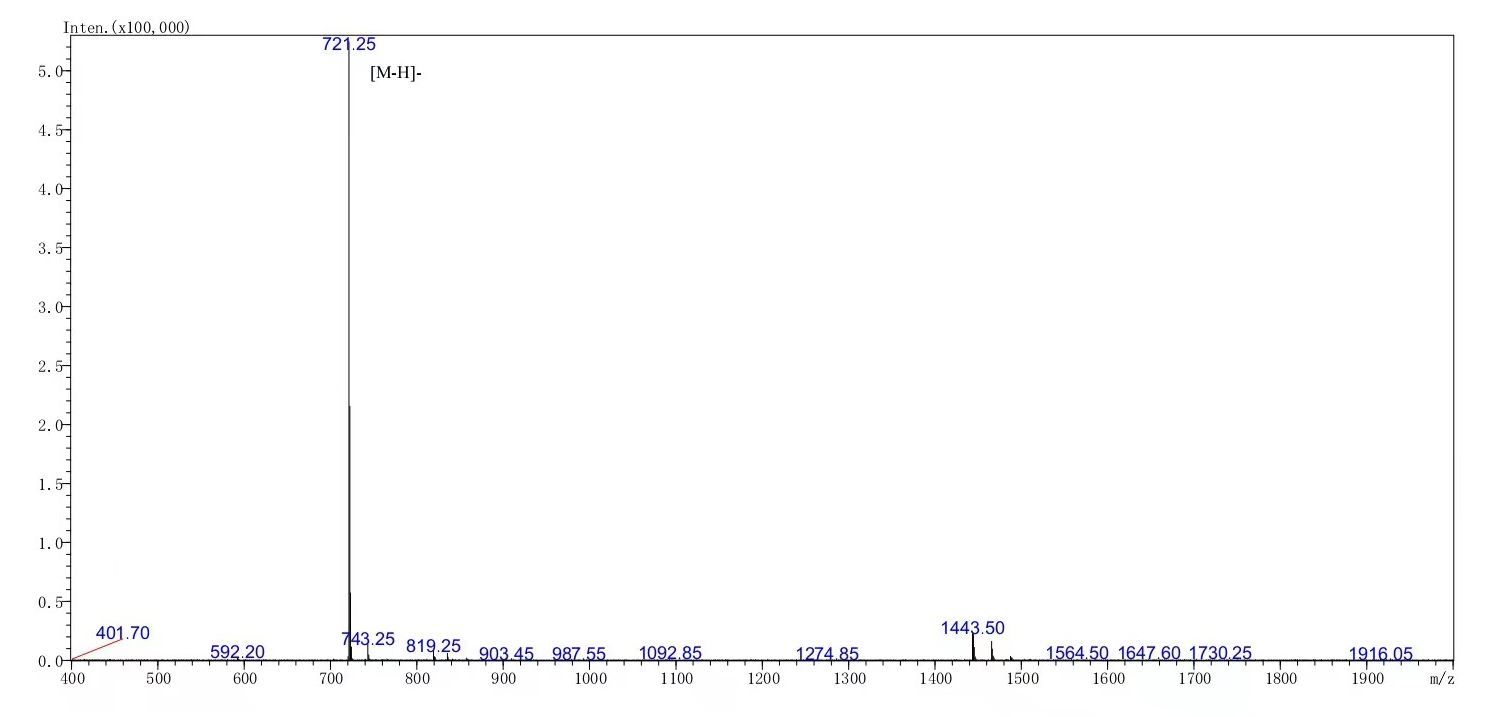


B

Figure S1. The HPLC chromatogram (A) and LC-MS spectrum (B) of synthetic peptide IEWEF.

Figure S2. The curve for the IC_50_ determination of synthetic peptide IEWEF.
